# Market Access Challenges and Solutions in Cell and Gene Therapy in The Netherlands

**DOI:** 10.3390/jmahp12030015

**Published:** 2024-07-30

**Authors:** Rimma Velikanova, Sharon Wolters, Hinko S. Hofstra, Maarten J. Postma, Cornelis Boersma

**Affiliations:** 1 Department of Health Sciences, University of Groningen, University Medical Center Groningen, Hanzeplein 1, 9713 GZ Groningen, The Netherlands; sharon@ascacademics.com (S.W.); m.j.postma@rug.nl (M.J.P.); c.boersma01@umcg.nl (C.B.); 2 Asc Academics, Hereweg 120, 9725 AK Groningen, The Netherlands; hinko.hofstra@ascacademics.com; 3 Department of Economics, Econometrics and Finance, University of Groningen, Antonius Deusinglaan 1, 9713 AV Groningen, The Netherlands; 4 Health-Ecore, Utrechtseweg 60, 3704 HE Zeist, The Netherlands; 5 Department of Management Sciences, Open University, Valkenburgerweg 177, 6419 AT Heerlen, The Netherlands

**Keywords:** cell and gene therapies, market access, time-to-patient access, health technology assessment (HTA), pharmaceutical policy

## Abstract

With the increasing pipeline of cell and gene therapies (CGTs) and the expected surge in the number of approvals, understanding the market access landscape becomes crucial for timely patient access. This study evaluates the challenges Dutch stakeholders encounter in CGT market access, offering insights for improving time-to-patient access. A traditional literature review was conducted to identify market access challenges and solutions for CGTs. Based on the findings, participants for semi-structured interviews, designed using an interview guide adapted to the Dutch context, were selected to capture diverse perspectives on market access. This review included 124 relevant articles out of 2449, covering several aspects of market access of CGTs. Subsequently, interviews with 16 stakeholders from academia, patient advocacy groups, manufacturers, health insurers, payers, hospital pharmacists, healthcare practitioners, and the Association of Innovative Medicines were conducted. Stakeholders identified challenges and proposed solutions for reimbursement package management, clinical trials, health economics, payment models, and procedural and organisational aspects. Thematic analysis revealed unique country-specific challenges and solutions in the Netherlands. This research provides insights into these challenges and potential solutions, emphasising the need for collaborative efforts among stakeholders to develop practical and multidisciplinary measures to improve the market access landscape for CGTs in the country.

## 1. Introduction

Despite the surge in the availability of cell and gene therapies (CGTs) over the past two decades, only a limited number of CGTs received reimbursement shortly after European Medicines Agency (EMA) approval. Nevertheless, considering a growing pipeline of products in clinical trials, the number of these approvals is anticipated to increase in the near future as up to 21 cell therapy launches and 31 gene therapy launches are expected by 2024 [1,2]. The main challenges for the market access procedure in Europe are due to differences between regulatory and reimbursement assessments along with the unique nature of CGTs and include dealing with limited patient populations, conducting long-term clinical effectiveness and safety evaluation, managing differences in assessment frameworks among countries, determining the value of health outcomes, estimating costs, selecting appropriate discount rates, incorporating equity considerations, and addressing affordability [3,4,5,6,7,8,9]. These issues are discussed in the international literature, which also offers recommendations on how the guidelines on economic evaluation methodology need to be adapted to assess CGTs [3,7,9,10,11,12,13,14]. Several health technology assessment (HTA) agencies have adapted their guidelines to address challenges specifically associated with CGTs, recommending increasing thresholds for severe diseases (Sweden and Norway), adding disease severity measurement through quality-adjusted life years (QALYs) shortfall (the U.K.), and introducing cure models with conservative and optimistic scenarios and durability of effect threshold analyses (Institute for Clinical and Economic Review in the U.S.) [15,16,17,18].

As the number of market access and reimbursement submissions for CGTs grows, stakeholders, including HTA bodies, payers, insurers, and healthcare professionals, face increased pressure to assess these innovative treatments. The variations in regulatory landscapes across different countries underscore the importance of mapping the opportunities and difficulties associated with CGT assessments at the national level. In the Netherlands, for instance, 40% of new (orphan) drugs are not reimbursed. Of particular concern within this subset is the fact that around 70% of orphan drugs with conditional or exceptional registration fail to secure reimbursement [19]. Furthermore, the median duration from EMA registration to hospital availability consistently exceeds 300 days for all three categories including the following: orphan drugs, drugs subjected to restricted distribution, and those that underwent conditional or accelerated marketing authorisation procedures. In the Netherlands, this timeframe has shown an annual increase, signifying a continuous trend toward heightened scrutiny [20]. The Dutch approach differs from that of other European nations such as Germany, Denmark, the U.K., Austria, and Norway, where orphan drugs are more actively reimbursed [21]. The Netherlands serves as an example of a country with a stringent regulatory approach. This was further emphasised by the new Dutch cabinet, which, after assuming office in January 2022, announced that costly medicines (i.e., medicines with an annual expenditure of EUR 20 million or more for one or more new indications) will not receive automatic coverage. Instead, they will undergo assessment and potential price negotiations before inclusion in the insured package [22]. Additionally, compared with other countries, the availability of CGTs in the Netherlands remains limited, with treatments like Tecartus, Abecma, Carvykti, Skysona, Zynteglo, Casgevy, and Hemgenix not yet accessible to Dutch patients as of June 2024 [23,24,25,26]. Consequently, access to treatment options for patients with respective conditions such as multiple myeloma, mantle cell lymphoma, cerebral adrenoleukodystrophy, beta-thalassemia, sickle cell disease, and haemophilia B remains constrained.

The primary objective of comprehending the challenges and solutions in the Netherlands is to enhance the time-to-patient access. This study is specifically designed to evaluate the obstacles faced by Dutch stakeholders in the market access of CGTs, and insights gained from this research have the potential to benefit other countries grappling with similar issues in the gene therapy field. Ultimately, the research findings inform recommendations for enhancing time-to-patient access in the Netherlands and neighbouring European countries.

## 2. Materials and Methods

### 2.1. Literature Review

**Figure 1:**
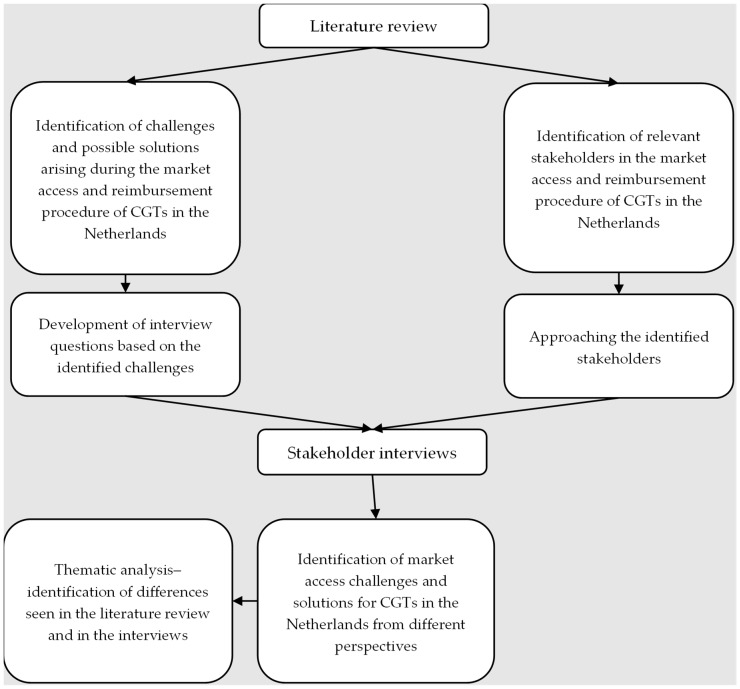
Methodology flowchart.

A pragmatic literature review was performed to identify market access challenges and solutions for CGTs. The traditional literature review method was chosen instead of a systematic review because it is better suited for qualitative research that aims to gain in-depth insights and generate hypotheses rather than test pre-determined ones [27]. This review included a grey literature search of HTA reports from the Dutch National Health Care Institute (ZIN; Dutch: Zorginstituut Nederland) and the National Institute for Health and Care Excellence (NICE) and other reports on trends in CGT market access in Europe, the U.S., and Canada. Based on the literature review, both the study sample and interview guide were further uncovered. Figure 1 depicts the methodology of this study in detail. The inclusion and exclusion criteria can be found in Appendix A Table A1.

### 2.2. Study Sample

The type of participants for the interviews was identified from the (grey) literature review and selected based on their experiences and significant expertise related to the market access of CGTs in the Netherlands, ensuring a well-rounded understanding of the challenges and solutions, providing diverse viewpoints on the subject, and ensuring conceptual saturation.

### 2.3. Developing the Interview Guide

The findings identified in the literature review informed the development of the interview guide designed to elicit in-depth responses from participants regarding their experiences, perspectives, and potential solutions related to market access, with a clear emphasis on the Dutch context. The interview guide was structured around open-ended questions. This approach provided a framework for exploring participants’ insights and capturing a comprehensive understanding of market access dynamics. The structure was designed to encourage participants to provide detailed and insightful responses while allowing them the flexibility to express their unique perspectives.

### 2.4. Administering Semi-Structured Interviews

Semi-structured, in-depth, qualitative interviews with participants were conducted from January to April 2022 through video conference calls. These interviews were recorded with participant consent, and open-ended questions were used to explore the participants’ experiences and perspectives on challenges related to market access of CGTs. The transcripts of the interviews were summarised to capture short and clear messages and were later validated and sent to each participant. The summaries provided a written version of the data that was analysed to better understand the participants’ experiences and perspectives on market access of CGTs.

### 2.5. Analysing Data

The thematic analysis of the transcribed interviews involved identifying recurrent themes and patterns in the participants’ experiences and perspectives related to market access of CGTs in the Netherlands [28]. Initially, the data were thoroughly familiarised by reading the transcripts. The text was highlighted, and data were systematically coded into relevant themes. These codes were subsequently grouped into broader categories. Each theme was clearly defined and named, with detailed descriptions developed to outline their key features. The categories were synthesised and interpreted to generate meaningful insights, examine their relationships, and address the research objective. The analysis also compared the solutions proposed by stakeholders to those identified in the literature, tabulating differences and similarities to highlight new solutions and identify those not yet implemented in the Netherlands. By analysing interview data to capture all relevant themes, conceptual saturation was achieved.

## 3. Results

### 3.1. Literature Review

**Figure A1:**
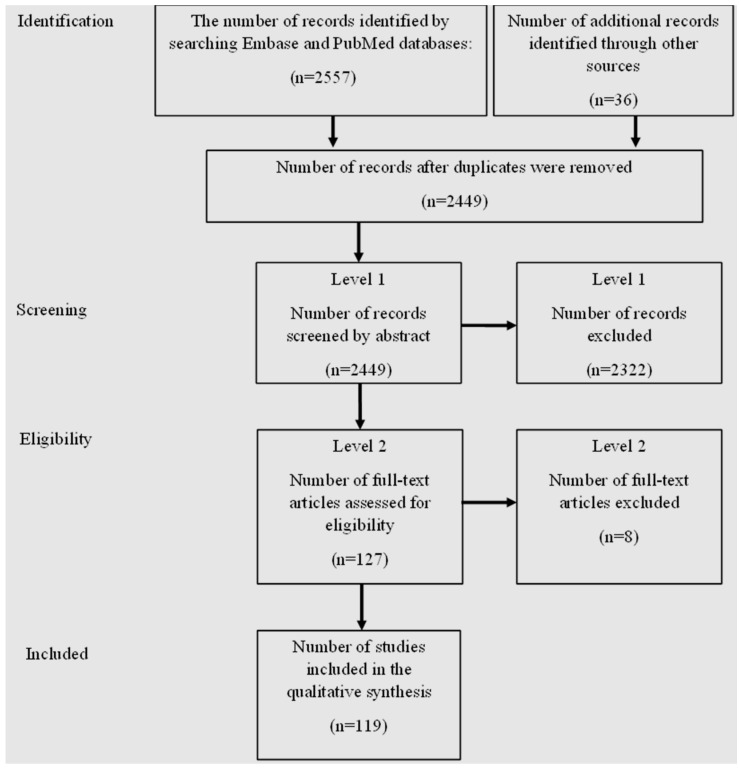
The PRISMA flowchart.

Out of the 2449 articles identified in the initial database searches, 124 relevant articles were selected for inclusion in this study. The following categories were identified in the literature review: clinical trial design, clinical evidence, health economics, reimbursement assessment, procedure, and organisation. The PRISMA flow diagram can be found in Figure A1 in Appendix A.

### 3.2. Study Sample

In total, 53 stakeholders were identified and contacted, of whom 16 agreed to the interview. There was an equal set of respondents from academia and patient advocacy groups, each accounting for 19% (three respondents), while manufacturers, health insurers, healthcare practitioners, and Association of Innovative Medicines members (Vereniging Innovatieve Geneesmiddelen, VIG) each represented 13% (two respondents) of the respondents. The lowest concentration was of hospital pharmacists and payers, representing 6% (one respondent) each.

### 3.3. Interview Guide

The interview guide focused on the following categories: reimbursement package, clinical trial design and assessment of the art and practice, health economics, payment models, procedure, and organisation. It can be found in Appendix A Table A2. The interviews lasted 45 min to one hour.

### 3.4. Views on the Reimbursement Package Management Challenges

Despite the benefits and facilitators of access, such as low budget impact and curative potentials, assessing CGTs presents several challenges, as acknowledged by all stakeholders in the interviews. Overall, 83% of respondents recognised the uncertainty in the long-term effectiveness of the therapy, 33% acknowledged the difficulty in comparing clinical data with standard care, 33% stressed the high burden associated with reimbursement procedure, 16% suggested there was a lack of designated centres of expertise and lack of clarity on whether the current reimbursement procedure is applicable for CGTs as those are therapies, not drugs, and another 16% highlighted the small patient populations and high prices of CGTs as considerable challenges to bear.

There was broad agreement on using RWE and long-term patient registries among academic HTA experts, the VIG, manufacturers, and health insurers. Both VIG and manufacturers suggested post-marketing reassessments based on phase IV studies and long-term data follow-up from clinical trials, respectively. Former payers advocated for the use of agreement mechanisms, emphasising that agreeing on Minimal Clinically Important Difference (MCID) should be a priority. Healthcare practitioners suggested permitting extrapolations based on mechanisms of action.

While the stakeholders acknowledged the difficulty in comparing clinical data with standard care, their approaches to addressing this challenge varied. The former payer emphasised the need to agree on MCID despite the inherent difficulty and offered the same advice for small patient populations in trials. Additionally, the payer recommended agreeing on a general level of uncertainty permitted by all the stakeholders. Health insurers recommended taking into account the additional costs of CGTs and comparing these to the total multi-year treatment costs of the current standard of care.

The stakeholders also stressed the high burden associated with the reimbursement procedure. Manufacturers suggested that ZIN should become more flexible. Healthcare practitioners recommended setting clear criteria for which patients should receive treatment and allowing health insurers to appoint Centres of Excellence (CoE).

Health insurers identified a lack of designated centres of expertise as a significant challenge. They suggested coming to an agreement with the relevant professional groups (beroepsgroep) to establish and designate these centres.

To address the high prices of CGTs, they suggested the development of new pricing models.

### 3.5. Views on the Clinical Trials, State-of-the-Art, and Science Challenges

The effectiveness and safety of CGTs are highly uncertain for a variety of reasons, according to all stakeholders. As much as 71% of the respondents suggested this is due to the relatively short-term duration of clinical trials, while another 57% argued that it is due to the single-arm study design. Additionally, 57% of the respondents mentioned that small patient populations in clinical trials contribute to this uncertainty. Finally, 29% mentioned that disease-specific and not generally applicable outcome measures also contribute to the overall uncertainty.

The stakeholders generally agreed on the importance of using real-world evidence (RWE) and long-term patient registries to address the short-term duration of clinical trials and the need for long-term data collection. Academic HTA experts, health insurers, and the VIG all advocated for using RWE and ensuring ongoing data collection through patient registries. Specifically, health insurers highlighted the necessity of using phase IV data and making it obligatory to track patients in registries, while VIG emphasised the legal enforcement of post-market authorisation studies. However, the stakeholders took differing approaches. Healthcare practitioners proposed permitting extrapolation based on the mechanism of action, offering a different perspective on managing the lack of long-term data.

To address the challenge posed by single-arm studies, the stakeholders agreed on the use of historical data and indirect comparators. Health insurers, VIG, and academic HTA experts all recognised the value of historical control groups, though health insurers noted the difficulty in achieving comparable populations due to different inclusion criteria. VIG further recommended the use of biomarkers alongside patient follow-ups to track disease progression and stressed the importance of informing patients. Academic HTA experts highlighted the feasibility of agreeing upfront on indirect comparators from the literature, a practice already in place in the Netherlands. The former payer suggested that HTA agencies could provide more detailed information on the requirements for indirect comparisons, emphasising the need for clearer guidelines.

To address the challenge posed by small patient populations, the stakeholders generally agreed on the need for robust study requirements and increasing sample sizes. The patient advocacy groups and VIG both emphasised the importance of more stringent study requirements and international collaboration to enhance sample sizes. They also highlighted the need for head-to-head studies with standard therapies to establish non-inferiority or superiority and for early dialogue between manufacturers and regulatory authorities to ensure trials meet necessary assessment criteria. Healthcare practitioners proposed outcome-based agreements as a solution to the issue of small patient populations, offering a different perspective on managing this challenge. Academic HTA experts suggested that allowing HTA agencies to provide input on study designs approved by the EMA would improve the relevance and comprehensiveness of the trials.

Lastly, the stakeholders agreed on the need for standardised outcome measures to address the challenge posed by disease-specific outcomes. The patient advocacy group called for the EMA/FDA to implement stricter requirements for similar outcome measures across different trials within the same indications. VIG underscored the issue by noting that current outcome measures are disease-specific and not generally applicable, implying the need for a more standardised approach

### 3.6. Views on Health Economics Challenges

All survey respondents identified challenges related to the health economics methodology specific to the Netherlands. Overall, 57% of those surveyed reported a lack of clarity regarding the appropriate use, indicating that if the drugs were in the trajectory of “appropriate use (in Dutch: “gepast gebruik”)”, it would be easier to monitor, collect data, and treat patients more effectively and cost-effective as a result. Another 57% of the respondents stated that traditional cost-effectiveness analyses (CEAs) did not represent CGTs, as they did not account for the unique costs or discounting methods required for CGTs. Additionally, 43% noted uncertainty in the long-term effects of CGTs, and 29% reported difficulty in accurately assessing the quality of life of CGT patients.

The stakeholders’ approaches to tackling the challenge related to appropriate use varied. Healthcare practitioners suggested collaborating with local HTA to establish MCID before national use and updating clinical guidelines. Health insurers proposed allowing professional associations to set up CoE and adhering to specific start criteria to assess patient suitability, particularly recommending a single CoE for small patient populations. The patient advocacy group emphasised good follow-up, clear criteria for starting treatment, and linking follow-up to a CoE for better patient assessment. The former payer advocated for using registries to improve appropriate use through ongoing monitoring and follow-up research.

Academic HTA experts recommended adapting CEAs to include specific elements of CGTs, such as hospital training and quality systems, to address the limitations of traditional CEAs. VIG suggested companies provide input on cost-effectiveness models, develop new discounting methods, and include cost-effectiveness analyses early in the process. Manufacturers stressed creating Diagnosis Treatment Combinations (DBCs; Dutch: Diagnose Behandel Combinaties) to cover administration, safety, and hospital stay requirements.

To address uncertainty in long-term effects, health insurers recommended adding conditional reimbursement or payment trajectories, and VIG recommended including cost-effectiveness in an early stage. To address the difficulty in accurately assessing QoL, the former payer advised monitoring patients extensively to establish accurate baselines and suggested setting up registries and CoEs for continuous long-term monitoring.

### 3.7. Views on Payment and Model Challenges

According to all interview respondents, there are issues related to payment and payment models for CGTs. High up-front costs were considered a significant challenge for 83% of the respondents, 67% mentioned that hospitals bear additional costs not included in the price of CGTs, 50% reported that it is difficult to establish a payment model as stakeholders cannot agree on MCID to reach reimbursement, and 17% stated that CGT patients are not evenly distributed among health insurers and can switch coverage after treatment. Additionally, a notable 17% of the respondents highlighted that flat discounts resulting from price negotiations are unsustainable because of their contribution to inconsistent pricing, lack of transparency, and fairness. Furthermore, this approach can create misaligned incentives, focusing on securing profitable agreements rather than addressing broader public health needs, thus impeding effective collaboration and partnership between pharmaceutical companies and governments.

Many of the stakeholders, including healthcare practitioners, the former payer, health insurers, the VIG, and manufacturers, advocated for various financial agreements to address this issue of high upfront costs. Different options for financial agreements included managed entry agreements with instalments proposed by health insurers, outcome-based payments or refunds for ineffective treatments suggested by healthcare practitioners and the former payer, and volume-based rebates recommended by manufacturers.

Additionally, health insurers recommend shifting phase III clinical research to real-life research to shorten registration times and reduce treatment costs. The former payer emphasised the need for agreement on outcome measures to make outcome-based agreements feasible.

To address the fact that hospitals bear additional costs that are not included in the price of CGTs, healthcare practitioners suggested including these additional costs in reimbursement. The former payer recommended covering and integrating these costs into DBCs and adjusting them accordingly. The VIG emphasised the need to standardise hospital requirements to treat patients with CGTs.

Regarding the challenge of agreeing on the MCID, the former payer proposed involving a third party, such as a professional association, to help set these thresholds and facilitate pricing agreements. They emphasised that outcome measures and MCID agreements should be linked to a patient registry and CoE. Academic HTA experts recommended using these payment models in combination with an early access program to increase availability and improve data collection, thereby reducing existing uncertainties.

To mitigate the uneven distribution of CGT patients among health insurers, health insurers proposed creating a joint fund to pay for CGT treatments, which would help distribute the financial burden more evenly.

Finally, to tackle the issue of unsustainable flat discounts resulting from price negotiations, health insurers suggested implementing risk-sharing and instalments to ensure more sustainable and transparent pricing models.

### 3.8. Views on Procedural and Organisational Challenges

According to the interview results, there are several challenges facing organisations, including bureaucratic and legislative issues. Specifically, 71% of the respondents reported that hospitals and academic groups face challenges related to legislation, certification, capacity, and equipment associated with CGTs. Academic centres must meet multiple manufacturers’ requirements and receive permission to work with CGTs, which is time-consuming. Pharmacists must fulfil requirements that are usually not feasible for a hospital pharmacist. Hospitals must purchase additional equipment and adhere to different quality control systems per manufacturer. In total, 57% of the respondents noted issues with the HTA process, such as extended time between marketing authorisation and market access, high costs due to separate reimbursement processes per country, lack of clear guidance on early access programs, and the absence of independent advice during ZIN assessment. Additionally, 29% of respondents cited challenges related to collecting RWE and low patient numbers due to low publicity and local data collection. Finally, 14% of the respondents mentioned that clinical guidelines are incomplete before or upon reimbursement.

To address the challenges related to legislation, certification, capacity, and equipment associated with CGTs, healthcare practitioners proposed creating a universal certificate/license for hospitals that covers all requirements and satisfies various manufacturers, preventing repeated assessments and audits. They also highlighted the DARE-NL platform, which ensures harmonised development, clinical testing, and sustained patient access through standardised practices across Dutch academic centres. The patient advocacy group suggested creating agreements in terms of accountability, spillage, and contracts, as well as arranging permission procedures per academic centre with a designated committee. Academic HTA experts recommended establishing a universal license/system that covers multiple products, encouraging manufacturers to fit their products into such a system. VIG emphasised the need for good coordination and upfront early dialogues to address the questions and needs of payers, healthcare practitioners, and patients, noting that they have already reduced the certification process time from one year to 28 days on paper.

Regarding the HTA process challenges, such as extended time between marketing authorisation and market access, high costs from separate reimbursement processes per country, the lack of clear guidance on early access programs, and the absence of independent advice during ZIN assessment, the stakeholders proposed various solutions. Health insurers suggested starting conversations before the product leaves the “lock” and arranging a central European process for registration and reimbursement. The patient advocacy group recommended clear discussions between hospitals and manufacturers about indications, finance, risks, insurance, and obligatory data collection during early access. They also emphasised the importance of proactive engagement with PAGs and allowing healthcare practitioners to comment earlier to align with PAGs. Furthermore, they called for academic centres to receive budget support for phase I and II research and to make it possible to register through the Central Committee on Research Involving Human Subjects only. The former payer questioned whether the responsibility should remain with private parties and suggested adding hospitals to the registration framework. Manufacturers recommended that ZIN should look at similar dossiers to expedite the assessment process.

To overcome challenges related to collecting RWE and low patient numbers due to low publicity and local data collection, the stakeholders proposed several solutions. Health insurers suggested setting up registries independently and on a European level to facilitate data collection. VIG recommended creating enthusiasm among healthcare practitioners and patients by explaining the treatment and seeking media attention to increase awareness. They also emphasised cooperation among healthcare practitioners to provide advice and pre-sort the designation of CoE, noting that often only one centre participates in trials in the Netherlands.

Lastly, to address the issue of incomplete clinical guidelines before or upon reimbursement, manufacturers proposed that professional associations should create better patient flows and estimations. They emphasised the importance of including new interventions in the guidelines before reimbursement.

### 3.9. Thematic Analysis of Interviews and the Literature Review

A thematic analysis of the challenges and recommendations identified in the literature review and interviews was conducted. In the context of the Netherlands, it was found that there were more country-specific challenges and solutions than those currently discussed in the literature. The detailed overview is presented in Table A3 and Table A4 in Appendix A. Notably, these include developing more robust data requirements as early as in dialogue with EMA. Establishing clear criteria for the CoEs is also critical to ensure these institutions are well-equipped to handle CGTs effectively.

Additionally, the stakeholders emphasised the importance of considering extra costs associated with CGTs, accounting for DBC costs. Integrating price negotiations and financial models early in the market access process was identified as a key strategy to mitigate potential delays and uncertainties. Furthermore, meaningful involvement of patient and clinician associations was underscored as essential for developing patient-centric and clinically relevant solutions.

Interestingly, the Dutch stakeholders did not mention certain recommendations discussed in the literature, such as increasing the willingness-to-pay threshold for health technologies like CGTs, integrating all elements in economic modelling using multi-criteria decision analyses, and establishing patient advocacy groups to advocate for the affordability and accessibility of CGTs.

## 4. Discussion

This paper sheds light on published challenges and solutions related to the market access of CGTs in the Netherlands, as seen from the stakeholders’ perspectives. This study conducted 16 interviews with stakeholders from various areas of expertise, including academia, patient advocacy groups, manufacturers, health insurers, healthcare practitioners, payers, hospital pharmacists, and VIG. The findings of this study summarise the stakeholders’ views on the readiness of HTA agencies to assess CGTs, long-term safety and effectiveness, health economics methodology, funding models, and other policies relevant to stakeholders. This article highlights common challenges all stakeholders face, such as uncertainty in long-term effectiveness, difficulties in comparing clinical data, high costs, lack of designated expertise centres, and small patient populations. Each stakeholder group has a different focus on addressing these issues, reflecting the complex dynamics and underscoring the need for a comprehensive discussion. The solutions to address the challenges include improving communication and collaboration, developing a regional strategy for RWE, establishing expertise centres, collecting RWE, establishing robust study requirements, developing new health economics methodologies, and introducing new payment models.

While this study offers a unique perspective, it has inherent limitations to its qualitative method. This study did not account for recent studies published after the literature review, and the reliance on spontaneous recall during interviews may affect the accuracy of the findings [6,14,29,30,31,32,33,34,35,36]. Although the data collection occurred a few years ago, the answers and our recommendations remain relevant because the common challenges are still faced and the proposed solutions continue to be necessary and applicable.

Despite these limitations, the consistent findings among different stakeholders strengthen the results and reduce the impact of recall bias. Although this study had a limited number of interviewees, which is common in qualitative research, the study design partly ensured the conceptual saturation of each stakeholder group when possible. Additionally, the number of respondents was deemed sufficient because of the high level of expertise and diverse perspectives each participant brought to this research. Their comprehensive knowledge and varied backgrounds ensured a rich, in-depth topic exploration. The findings of this study add to the numerous articles published on the topic, as they not only highlight the challenges faced by the stakeholders in the field but also present specific recommendations for improving market access of CGTs derived from the stakeholders’ interviews. Unlike many other articles that only address a single perspective and lack recommendations for collaboration among stakeholders, this study provides a comprehensive view of the situation in the Netherlands from different angles and perspectives. It emphasises the importance of a united approach to improving market access. It is important to note that while these findings are primarily applicable to the Netherlands, they can be relevant to countries with similar regulatory environments, such as Belgium, Ireland, and Luxembourg. It is recommended that future research initiate similar studies in other European countries to identify corresponding vulnerabilities and find opportunities for pan-European collaboration.

## 5. Conclusions

This research provides a novel exploration of market access challenges and solutions for CGTs in the Netherlands by integrating diverse stakeholder perspectives through comprehensive qualitative interviews, presenting realistic and achievable recommendations. It emphasises the importance of collaborative efforts among stakeholders in the coming years to mitigate these challenges and develop comprehensive multidisciplinary solutions. This manuscript could yield a call to action to initiate the process of creating tangible action plans to address these issues, providing a united starting point for all stakeholders to work together in improving patient access to CGTs in the Netherlands.

## Data Availability

All the relevant data are provided in this article.

## Appendix A

**Table A1:** PICOS criteria for the inclusion and exclusion of studies.

Category	Inclusion Criteria	Exclusion Criteria
Population (P)	Any patient population that is eligible for CGT	Patient population not eligible for CGT
Intervention (I)	CGTs	Not CGT
Comparators (Cs)	Any	None
Outcomes (Os)	Challenges and solutions in CGT market access	Not discussing CGT market access challenges and solutions
Study design (S)	Any	None
Language	English or Dutch language	Non-English or non-Dutch language
Time limit	Published from 2016 to July 2021 ^1^	Studies published before 2016
Country	Any	None

CGT indicates Cell and Gene Therapy; PICOS indicates population, intervention, comparators, outcomes, study design, language, time limit, and country. ^1^ This period captures key regulatory approvals, reimbursement challenges, and early market experiences of gene and cell therapies. It includes the latest data and trends, reflecting the evolving market access landscape.

**Table A2:** Interview guide.

Questions by Category
Introduction: We are conducting research on the market access challenges of CGTs in the Netherlands. The lead time for CGT medications is long due to additional challenges, and we hope this research will help streamline and accelerate the market access process. We aim to contribute to the structural access of patients to CGTs through these interviews. We have already identified a number of challenges in the literature, but we would like to identify the key challenges and potential solutions experienced by various parties involved in the Dutch practice through these interviews. We hope this will contribute to a future-proof package management as mentioned by ZIN to keep innovative care high quality, accessible, and affordable in the future. Finally, we would like to discuss possible solutions for the challenges, the transformations needed for this, and what the current state of affairs is.
Questions by Category
Open Questions
How have you been involved in the (reimbursement) processes to make a CGT therapy available for a patient? What was your role in this process?
2. What innovation do you believe CGTs can bring? What are the benefits of CGT for the patient? What does this mean for [stakeholder X]?
3. What challenges (in order of importance) exist in making CGTs available based on your experience?
4. {if not spontaneously mentioned in 2, focus on reimbursement process} What specific challenges exist for CGTs that complicate the reimbursement process?
For the rest of the interview, we have divided the questions into the following topics: clinical CGT research, current state of science and practice, health economics, future-proof package management, funding, and procedures/organization.
Clinical Trial Challenges
Current clinical CGT studies have a number of shortcomings.
CGT studies are often small (indicated for orphan diseases). How can uncertainties regarding effectiveness and safety be further substantiated? What criteria must the data meet to tentatively conclude that the therapy has added value?
2. How should we deal with the lack of/limited clinical (long-term) data? What are possible alternatives/conditions for this?
3. CGT trials are often short in relation to the expected long-term outcomes. How can we reduce the uncertainty that this creates?
4. We regularly see the use of non-clinically relevant/non-validated/secondary/surrogate endpoints in clinical studies. How can this be prevented? If it cannot be prevented, how can it be managed?
5. RCTs are not always possible for CGTs. How can we still obtain comparative evidence that sufficiently reduces uncertainty? How is this handled in the assessment of the state of science and practice? Should certain conditions (collection of RWE, pay for performance) be met?
State of Science and Practice
CGTs obtain reimbursement through the same process as other therapies (conditional admission process or standard assessment from the sluice)
What are the main challenges for CGTs regarding HTA within the current assessment framework?
2. Is the assessment framework being adjusted and why (which elements are currently not suitable and should ideally be adjusted specifically for CGT in the future)? Is ZIN working on this (timelines)?
3. Is there use of core outcome sets for CGTs in the Netherlands? Is their use advisable?
4. Given the burden of proof for CGTs; what conditions can be applied to allow these CGTs with limited evidence to still be included in the insured package? How are patient registries in the Netherlands linked to conditional reimbursement and/or pay-for-performance? (e.g., Luxturna)
Health Economics
What problems are we currently seeing in the cost-effectiveness analysis of CGTs?
2. Does the current methodology of CEA sufficiently align with the assessment of the cost-effectiveness of CGT?
3. What are the main challenges in determining the quality of life of patients treated with CGTs and how can these challenges be addressed?
4. What are the benefits of CGTs that are currently not captured in traditional cost-effectiveness models? How can these be included in the future?
5. What are the main challenges in determining the appropriate discount rate and time horizon in CGT cost-effectiveness models?
6. How should cost-effectiveness models deal with the lack of long-term data?
7. How can HTA bodies/insurers handle uncertainty in the area of cost-effectiveness?
Future-Proof Package Management
What impact will the arrival of CGTs have on future-proof package management? How can we anticipate this? Where are the current bottlenecks in package management? Is it desirable to set up a separate route to reimbursement for CGT? A separate assessment framework? What would such a route look like according to you? Similarly, the assessment framework?
2. What are important best practices that have worked well in making CGT available (e.g., in bringing parties together)?
3. How early should a manufacturer start involving every party (ZIN, doctors, insurers, patient organizations) in the assessment process?
4. How do you see the collaboration with and the role of manufacturers? How could this be improved?
Funding
CGTs are associated with high upfront costs.
Are there CGT-specific challenges in terms of funding? (add-on experience, can all care activities be claimed in DBC or is a modification needed, other funding issues related to the high one-time price) What is the impact of such a bottleneck? How can this bottleneck be prevented/resolved? [Example if not mentioned: How should we deal with the costs of additional care activities not covered by the current DBC/DOTs?]
2. In your opinion, what is the best way to handle the high upfront costs?
Various innovative payment models can be used to reduce uncertainties/risk sharing or to spread payment over time.
3. How can such payment models be deployed to meet the challenges in the context of CGT? What is your experience with such payment models?
What is the current state of affairs regarding the development of payment models that better fit CGT? (this is referred to in the July 2021 letter to parliament about future-oriented package management).
4. Does ‘pay-for-performance’ have the preference, or are there more suitable payment models for the Dutch market?
5. What are important conditions that such a model must meet? [conditions for success]. How are the conditions for a pay-for-performance determined? What does this look like? Start-stop criteria, duration, etc.
6. How can we promote the appropriate use of CGTs?
Procedural and Organizational Challenges
Are there also other procedures besides the reimbursement procedures that affect the availability of CGT (separate regulations for CGT)?
2. Are there learnings from accelerating the availability of COVID-19 vaccines?
Closing
How is the collaboration between pharmaceutical companies and your organization regarding CGT? How could this be improved? What advice do you have for manufacturers to bring a CGT to the patient more quickly?
2. What are your main responsibilities in the process to ensure that patients quickly gain access to a CGT and how can the manufacturer further support you [include this as a second question]?

CGT indicates Cell and Gene Therapy; ZIN indicates Zorginstituut Nederland (Dutch National Health Care Institute); RCT indicates Randomized Controlled Trial; RWE indicates real-world evidence; HTA indicates health technology assessment; DBC indicates Diagnose Behandel Combinatie (Diagnosis Treatment Combination); DOT indicates DBCs on their way to transparency; CEA indicates cost-effectiveness analysis.

**Table A3:** Challenges identified from the literature review versus interviews.

	Challenges	Clinical Trial	Clinical Evidence—SvWP/Pakketbeheer	Health Economics	Payment Models	Value Assessment	Procedures & Organizational
TLR/ Dutch Stakeholders							
Overlapping challenges in TLR and Dutch stakeholders		- Mostly single-arm—short-term trials with a small number of patients	- Uncertainty in long-term efficacy and safety data - Lack of comparative data - High burden on the reimbursement package - Poor selection of study endpoints	- Uncertainty in long-term effectiveness and cost-effectiveness (wide ICER intervals) - High upfront costs not fitting the current payment structure - Current discounting on costs cannot be used	- High upfront cost for a (mostly) one-off treatment that introduces significant price pressure that needs new contracting approaches	- Questionable whether CGT can be valued within the current drug appraisal system	- Strict regulatory requirements for CGT and adaptations impacting developers - Pressure on hospitals for extra training/certificates - Extra costs on top of CGT for hospitals
Additional Dutch challenges derived from interviews			- No clarity on the assignment of CoEs for CGT - The small patient population makes it difficult to identify the eligible patient population - No consensus on endpoints or MCID per endpoint for RWE collection - No accurate HRQoL data are available	- Currently no incentives for appropriate use of CGT (inc. spillage) - Not all value components or additional costs are considered in the current CEA appraisal	- New DRGs or adjusted DRGs are necessary to cover all hospital costs related to CGTs - Flat discounts are not durable in CGTs for HICs - Payment models are difficult to set up - Rare diseases are not evenly distributed over jurisdictions of HICs - Patients can switch to other HIC after a one-off Tx	- Rigid and strict requirements by ZIN in comparison with EMA - Delays in ZIN assessment due to consultations at a late stage - Long time between market authorization and market access - Missing early dialogue	- Hospitals have an extra risk due to non-existing DRGs or not covering all costs for CGT - Hospital exempt rule is not clear yet - No owner of CGT problem - Collection of RWE data is difficult because of the low number of patients in NL - Environmental license needed for CGT

TLR indicates literature review; SvWP indicates Stand van de Wetenschap en Praktijk (State of Science and Practice); CoEs indicates Centers of Excelence; CGT indicates cell and gene therapy; ICER indicates Incremental Cost-Effectiveness Ratio; DRGs indicates Diagnosis-Related Groups; HICs indicates health insurers; CEA indicates cost-effectiveness analysis; RWE indicates real-world evidence; HRQoL indicates Health-Related Quality of Life; MCID indicates Minimal Clinically Important Difference; EMA indicates European Medicines Agency; ZIN indicates Zorginstituut Nederland (Dutch National Health Care Institute). Note: Red highlights indicate significant CGT challenges that are complex to overcome under current HTA and market access requirements in the Netherlands; amber challenges can be addressed with additional data or structural changes, while green challenges are more easily resolved through collaboration with stakeholders, focusing on procedural or organizational issues.

**Table A4:** Solutions identified from the literature review versus interviews.

	Solutions	Clinical Trial	Clinical Evidence—SvWP/Pakketbeheer	Health Economics	Payment Models	Value Assessment	Procedures and Organisational
TLR/ Dutch							
Overlapping Proposed Solutions in TLR and Dutch stakeholders		- Early interaction with regulators and HTA agencies on trial design and endpoints. - Ensure a uniform way of reporting CGT trials in a disease area. - Collect additional data in the real-world setting and use existing registries.	- Create a core outcome set to ensure all CGT trials in a disease area have the same comparable outcomes. - Use RWE in (re) assessment.	- Include scenarios with different discounting rates.	- Implement payment models/MEAs.	- Develop new value frameworks for CGT. - HTA assessors should be more flexible and reactive to CGT-specific challenges - Share knowledge	- Regulators should be flexible.
Proposed solution in TLR			-	- Increase WTP threshold for Tx as CGT. - Integrate all elements in economic modelling like MCDA.	- PAGs can advocate for the affordability and accessibility of CGT.		
Additional Dutch proposed solutions		- More robust data requirements needed by EMA.	- Ensure clear criteria for designation of CoE(s). - Early interaction with HTA agency and agree upfront on indirect comparator or historical data to be used. - Set clear criteria for EPP. - Use same outcomes during re-assessment as during initial assessment and define MCID beforehand. - Extrapolation of effectiveness and safety data based on MoA should be allowed. - Ensure a long-time FU for HRQoL in registries by CoE.	- Take additional costs related to CGT into account in a CEA and compare them to multi-year treatment costs - Develop a different way of discounting.	- Acknowledged by MoH that payment models and conditional approval are complex but will be investigated. - Various contracting agreements could be considered. - Start negotiations on price and payment models sooner. - Create a joint fund for CGT across HICs - Use the payment models also in combination with early access programmes.	- The assessment framework can be adapted but will not take away the uncertainty - Questionable whether CGT should fit in drug. assessment - Involve clinician association in assessment (patient flow, update guideline, positioning CGT). - ZIN should become faster in assessing dossiers.	- Early dialogue and coordination among all stakeholders - Clarify the hospital exemption rule - PAG indicates that they can play an important role for MNF and ZIN - PAGs can raise awareness and cultivate enthusiasm with HCP and patients for trials and RWE collection (inter)nationally via social media. - Ensure new/adjusted DRGs are developed to cover all costs - Create a universal certificate/license for administering CGT in hospitals/CoE(s) - Use a platform to share knowledge among CoEs.

TLR indicates Literature Review; SvWP indicates Stand van de Wetenschap en Praktijk (State of Science and Practice); HTA indicates Health Technology Assessment; CGT indicates cell and gene therapy; RWE indicates real-world evidence; CoE(s) indicates Centre(s) of Excellence; EMA indicates European Medicines Agency; WTP indicates Willingness to Pay; Tx indicates Treatment; MCDA indicates Multi-Criteria Decision Analysis; CEA indicates cost-effectiveness analysis; MoA indicates the mechanism of action; FU indicates follow-up; HRQoL indicates Health-Related Quality of Life; PAG indicates patient advocacy group; MoH indicates Ministry of Health; HICs indicates health insurance; MNF indicates manufacturer; ZIN indicates Zorginstituut Nederland (Dutch National Health Care Institute); HCP indicates Health Care Provider; EPPs indicates Early Patient Programs; DRG indicates Diagnosis-Related Group. Note: Red highlights indicate significant CGT challenges that are complex to overcome under current HTA and market access requirements in the Netherlands; amber challenges can be addressed with additional data or structural changes, while green challenges are more easily resolved through collaboration with stakeholders, focusing on procedural or organisational issues.
